# Community analysis of bacteria colonizing intestinal tissue of neonates with necrotizing enterocolitis

**DOI:** 10.1186/1471-2180-11-73

**Published:** 2011-04-12

**Authors:** Birgitte Smith, Susan Bodé, Bodil L Petersen, Tim K Jensen, Christian Pipper, Julie Kloppenborg, Mette Boyé, Karen A Krogfelt, Lars Mølbak

**Affiliations:** 1 Statens Serum Institut, Artillerivej 5, 2300 Kbh. S, Denmark; 2 National Veterinary Institut- DTU, Bülowsvej 27, 1790 Copenhagen V, Denmark; 3 Neonatel Department 5023, Rigshospitalet, Blegdamsvej, 2100 Kbh. Ø, Denmark; 4 Pathologic Institut, Rigshospitalet, Blegdamsvej, 2100 Kbh. Ø, Denmark; 5 Faculty of Life Sciences, University of Copenhagen, Bülowsvej 17, 1870 Frb., Denmark

**Keywords:** FISH, laser capture microdissection, microbiota, necrotizing enterocolitis, pneumatosis intestinalis, *Ralstornia*

## Abstract

**Background:**

Necrotizing enterocolitis (NEC) is the most common gastrointestinal emergency in newborn neonates. Bacteria are believed to be important in the pathogenesis of NEC but bacterial characterization has only been done on human faecal samples and experimental animal studies. The aim of this study was to investigate the microbial composition and the relative number of bacteria in inflamed intestinal tissue surgically removed from neonates diagnosed with NEC (n = 24). The bacterial populations in the specimens were characterized by laser capture microdissection and subsequent sequencing combined with fluorescent in situ hybridization (FISH), using bacterial rRNA-targeting oligonucleotide probes.

**Results:**

Bacteria were detected in 22 of the 24 specimens, 71% had moderate to high densities of bacteria. The phyla detected by 16S rRNA gene sequencing were: *Proteobacteria *(49.0%), *Firmicutes *(30.4%), *Actinobacteria *(17.1%) and *Bacteroidetes *(3.6%). A major detected class of the phylum *Proteobacteria *belonged to *δ-proteobacteria*. Surprisingly, *Clostridium *species were only detected in 4 of the specimens by FISH, but two of these specimens exhibited histological pneumatosis intestinalis and both specimens had a moderate to a high density of *C. butyricum *and *C. parputrificum *detected by using species specific FISH probes. A 16S rRNA gene sequence tag similar to *Ralstonia *species was detected in most of the neonatal tissues and members of this genus have been reported to be opportunistic pathogens but their role in NEC has still to be clarified.

**Conclusion:**

In this study, in situ identification and community analysis of bacteria found in tissue specimens from neonates with NEC, were analysed for the first time. Although a large variability of bacteria was found in most of the analyzed specimens, no single or combination of known potential pathogenic bacteria species was dominating the samples suggestive NEC as non-infectious syndrome. However there was a significant correlation between the presence of *C. butyricum *&*C. parputrificum *and histological pneumatosis intestinalis. Finally this study emphasizes the possibility to examine the microbial composition directly on excised human tissues to avoid biases from faecal samples or culturing.

## Background

Necrotizing enterocolitis (NEC) is an acute inflammatory disease that affect the intestinal tract of neonates [1]. It remains one of the most common gastrointestinal emergencies in newborn neonates [2]. Onset of NEC occurs within the first three months of life and neonates who are of low birth weight and under 28 week gestation are the most susceptible [3]. The ileum and the proximal colon are the frequently affected although any segments of the gastrointestinal tract can be involved [4]. The course of NEC is multifactorial and the most important elements is prematurity, enteral feeding, bacterial colonization and an inappropriate pro-inflammatory response [5]. It is believed that immaturities of these functions due to age predispose the premature infant to intestinal injury and inappropriate responses to injury.

The bacterial role in NEC still needs to be clarified. Suggestions such as an imbalance of the gastrointestinal microbiota, overgrowth of potential pathogenic bacteria, and ischemia causing mucosal lesions that gives the bacteria systemic access have been followed but so far no specific pathogens have been identified. Correlation of NEC with bacteria has been suggested by analysing faecal samples, however, this analysis of faecal samples is often far from the affected site and may not be representative [5-11]. The use of formalin-fixed paraffin-embedded tissue samples give an opportunity to investigate a unique stock of archival disease-specific material. The method is challenged to access the limited and fragmented bacterial DNA present in the tissue. To characterize the bacterial population in the formalin-fixed NEC tissue laser-capture-micro-dissection (LCM) combined with fluorescence in situ hybridization (FISH), using a bacteria ribosomal RNA (rRNA)-targeting oligonucleotide probe, was used [12]. The bacterial 16S rRNA gene was PCR amplified and sequenced by pyrosequencing. The bacterial distribution was verified and visualized within the lumen and mucus of the intestinal tissues with fluorescent in situ hybridization (FISH) with group and species specific probes targeting individual microbial cells (Table 1). The aim of this study was to investigate the microbial composition and the relative number of bacteria in affected intestinal tissue samples surgically removed from neonates diagnosed with NEC and to relate this with the patient data such as antibiotic treatment.

**Table 1:** Target for probes and sequences of rRNA-targeting oligonucleotide probes and 16S rRNA gene targeting primers used in this study

Targets for probes	Oligonucleotide Probes	Sequence	Fluorophor	Reference
** Bacteria**	S-D-bact-0338-a-A-18	5' GCT GCC TCC CGT AGG AGT 3'	Fluorescein	[37]
**Enterobateria**	GAM42a	5' GCC TTC CCA C(AT)TCGT TT 3'	Fluorescein	[38]
	S-S-C.perfring-185-a-A-18	5'TGG TTG AAT GAT GAT GCC 3'	Cy3	[21]
***Clostridium *spp.**^**1**^	S-S-C.paraputri-181	5' CAT GCG AAC GTA CAA TCT 3'	Cy3	This study
	S-S-C. butyricum-663	5'AGG AAT TCT CCT TTC CTC 3'	Cy3	This study
	S-S-C.diff-193-a-A-18	5'TGT ACT GGC TCA CCT TTG 3'	Cy3	[21]
**Actinobacteria**	pB-00182	5'TA TAG TTA CCA CCG CCG T 3'	Cy3	[39]
**Lactobacillus & Enterococcus**	Lab158	5'GGTAT TAJ CAY CTG TTTCCA3'	Cy3	[40]
**Bifidobateria**	pB-00037	5'CC AGT GGC TAT CCC TGT GTG AAG G3'	Cy3	[41]
	**PCR Primers**			
**Bacteria**	Bact64f	5'-CY TAA YRC ATG CAA GTC G-3'		[42]
**Bacteria**	Bact109r1	5'-YY CAC GYG TTA CKC ACC CGT-3'		[42]
**Bacteria**	PyroBact64f	5'-CAT GCA AGT CG-3'	Biotin C-6	This study

^1 ^The Clostridium probe is a mixture of four clostridium species: *C. perfringens, C. difficile, C. butyricum *and *C. parputrificum*

## Result

Twenty-four neonates with different gestational age were enrolled in this study because they all had intestinal tissues surgically removed. Sections from the small intestine were removed in 15 neonates, from both the small intestine and the large intestine for 6 neonates, and only from the large intestine in 3 neonates. Eight of the 24 neonates died but there was no correlation between NEC-score and death. All data have been described in Table 2, but in summary three neonates were full-term; two of these had heart disease and one foeto-maternal bleeding. Three neonates were small for gestation. Nine neonates had pneumatosis intestinalis and 11 neonates had free air in the stomach as observed by x-ray. For 21 of the neonates information regarding enteral feeding was available. Mothers' breast milk or bank milk was introduced between day 1 and day 5, and supported with either 5% or 10% glucose. If the neonate was not able to reach the level of enteral feeding after day 5, support by paraenteral nutrition was initiated; median 8 day SD 8.9 (n = 13). All neonates were treated with antibiotics for different time spans before the surgery (Table 3). The standard treatment for children <7 days was i.v. injection of ampicillin, gentamicin and metronidazole; standard treatment for children >7 days was i.v. injection of cefuroxim, gentamicin and metronidazole. The antibiotic treatment will influence the general bacterial colonization but to the best of our knowledge there is no study about how it influences the bacterial composition and load of the NEC affected intestinal tissues in humans.

**Table 2:** Clinical characteristics of the hospitalized neonates in this study

Characteristics	
**Mother**	
Antiboitics during labor, n (%)	3 (13)
Betamethasone, n (%)	14 (58)
**Neonate**	
Mode of delivery (caesarean section), n (%)	14 (58)
Sex (m), n (%)	13 (54)
Number of twins, n (%)	7(29)
Gestational age (weeks), median (95% confidensceinterval)	29 (25-40)
Gastational weight (g) median (95% confidensceinterval)	1030 (600,-3660)
Small for gastational age n (%)	3 (13)
APGAR	
1 min (median) n = 19	8
5 min (median) n = 20	10
Arterial cord pH	7.18
RDS treated with surfactant, n (%)	8 (33)
CPAP (median, SD) n = 20	4.5 (9.8)
Ventilatior days (median, SD) n = 18	4 (12.6)
Ischemems event^1^, n (%)	11 (46)
Ex-ray	
pneumatosis entestinalis	9
free air in the stomach	11
Operation day (median, range)	10.5 (3, 52)
Removed tissue	
small intestinal	15
small intestinal and large intestine	6
Large intestine	3

^1^Ischemic event: defined as one or more of this condition; perinatal asphyxia, polycythaemia, cyanotic congenital heard disease, patent ductus arteriosus, medication that suppress mesenteric blood flow, maternal preeclampsia

**Table 3:** Fluorescent in situ hybridization (FISH) scores on intestinal specimens from 24 NEC patients.

Patient number	Tissue	**Days of antibiotic**^**5**^	NEC score	EUB338	Enterobateria	**Clostridium**^**1**^	Actinobactere	Lactobacillus	Bifidobateria
25	small intestinal	3	14	0	0	0	0	0	0
26^4^	small intestinal	4	13	0	0	0	0	0	0
9^4^	small intestinal	1	10	1	1	0	0	0	1
2	small intestinal^2^	1	15	1	0	0	2	0	0
6	small intestinal	17	11	1	2	0	0	2	0
8	small intestinal	1	12	1	0	0	0	0	0
12	large intestinal	5	17	1	0	0	2	0	0
14	small intestinal	15	13	1	1	1	1	2	0
15	small intestinal^2^	5	19	1	0	0	2	0	0
16^4^	small intestinal	4	6	1	1	1	1	0	0
27	small intestinal	4	8	1	1	0	0	0	0
1	small intestinal	6	5	2	2	0	1	2	0
3^3^	large intestinal	1	11	2	2	2	2	0	2
7	small intestinal	5	13	2	2	0	1	0	0
10^4^	small intestinal^2^	4	13	2	1	0	0	2	0
11^4^	small intestinal	7	7	2	1	0	0	2	0
17	small intestinal	11	15	2	2	0	0	1	0
18^3^	small intestinal^2^	12	15	2	2	1	1	0	1
19	small intestinal	4	19	2	2	0	1	1	0
20	small intestinal	11	13	2	1	0	1	2	0
21	small intestinal^2^	2	12	2	2	0	1	0	0
22^4^	small intestinal	1	13	2	2	0	1	0	0
23	small intestinal	4	15	2	2	0	0	0	0
24^4^	large intestinal	2	13	2	2	0	1	0	0

The score was: 0: few bacteria;1: moderate number of bacteria; 2: high number of bacteria.

^1 ^The Clostridium probe is a mixture of four specific probes targeting Clostridium species: *C. perfringens, C. difficile, C. butyricum *and *C. parputrificum*

^2 ^The neonates had tissues from both the small intestine and large intestine removed but FISH analysis was only done on the small intestinal tissues

^3 ^Pneumatosis intestinalis verified by histopathology

^4 ^Dead after the surgical operation

^5 ^Before NEC diagnose

### Detection of bacteria in tissue samples by fluorescent in situ hybridization (FISH)

**Figure 1:**
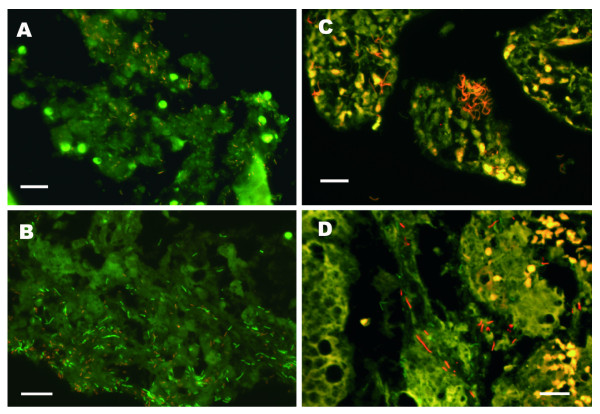
**Epifluorescence micrographs of fluorescent in situ hybridized tissue samples taken from neonates diagnosed with necrotizing enterocolitis**. All the specimens were hybridized with a general bacterial probe (EUB338) tagged with fluorescein (green colour) and group specific probes tagged with Cy3 (red colour). **A) **Visualization of Enterobacteria (GAM42a). **B) **Visualization of Actinobacteria ( pB00182). **C) **Visualization of *Clostridium butyricum *( S-S-C. butyricum-663) in the two neonates where pneumatosis intestinalis was verified by histopathology. **D) **Visualization of *Clostridium perfringens *(S-S-C.perfring-185-a-A-18) in neonate number 3 with pneumatosis intestinalis.The scale bar is 20 μm in all the micrographs.

Bacteria were detected in 22 of the 24 examined specimens, and of these 71% had a moderate to a high density of bacteria (Table 3). In 17 (70%) of the 24 specimens *Enterobacterieceae *were detected by a group specific FISH probe (Figure 1a) and a significant correlation was seen between this hybridization and the general bacterial probe based on the scoring system (p = 0.02).

In 4 specimens *Clostridium *species were detected by using a mixed *Clostridium *spp. probe targeting *C. perfringens, C. difficile, C. butyricum *and *C. paraputrificum*. Two of those specimens were by histological examinations observed to exhibit pneumatosis intestinalis and a significant correlation (p < 0.05) was found with the presence of the *Clostridium *spp even though the sample numbers are very small. In these two specimens *C. butyricum *and *C. parputrificum *were detected in high densities (Figure 1c), *C. perfringens *was detected in one of the specimens (figure 1d) whereas *C. difficile *was not detected in any of the slides. Nevertheless, no correlation was found between diagnosed neonates with pneumatosis intestinalis by x-rays and the specimens colonised with *Clostridium *spp. Finally, there was no correlation between the presence of bacteria by FISH and NEC score, type of nutrition, antibiotic usage, or death.

### Characterisation of bacterial composition in tissues removed surgically from neonates with NEC

**Figure 2:**
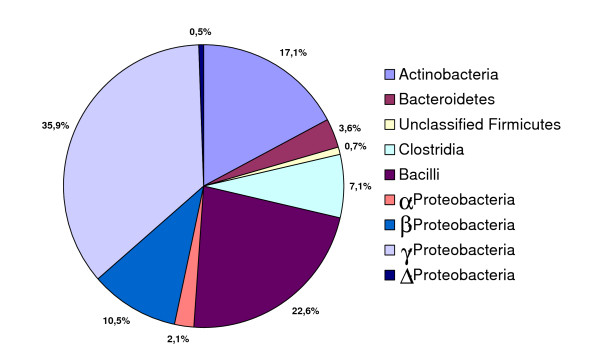
**The total bacterial composition from eight intestinal tissue samples by 16S rRNA gene clone library**. The γ-Proteobacteria dominated the total bacterial composition whereas the class Clostridia only accounted for a total of 7.1%

**Figure 3:**
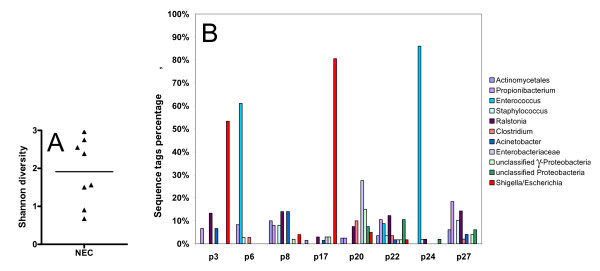
**Overview and diversity of the bacterial composition by clone library analysis**. a) Shannon's diversity index on phylum level divided the NEC infants in two groups. This difference could not be explained by antibiotic treatments or the severity of the necrotizing enterocolitis b) The bacterial 16S rRNA gene composition from each of the eight necrotic intestinal tissue samples. Bacterial groups whose abundance were more than 10% in any sample are shown as bars. *Enterococcus *and *Escherichia *spp. were the most abundant in the samples with a low Shannon diversity index where *Ralstonia *sp. was the most frequent group of species in the samples with a high Shannon index.

Eight neonates were selected for further characterisation of the bacteria located in the lumen and mucus layer of the inflamed tissues. Four of these neonates had received antibiotics for less than two days while the other four neonates had received antibiotics more than 10 days. A 16S rRNA gene library from each specimen was constructed. The individual tags (N = 364) were assigned to the closest mono-Phylogenetic group in order to obtain a Phylogenetic classification. In total, 41 consensus tags were identified (Table 4). The frequencies of 16S rRNA gene sequences from all specimens were grouped according to their overall phylogeny and the phyla were *Proteobacteria *(49.0%), *Firmicutes *(30.4%), *Actinobacteria *(17.1%) and *Bacteroidetes *(3.6%) (Figure 2). *δ-proteobacteria *was the major detected class of the phylum *Proteobacteria*. The Shannon diversity index was calculated based on the total library cloning sequences for each neonate (Figure 3). The Shannon diversity index revealed two distinct groups. The neonates p3, p6, p17 and p24 clustered together with a low Shannon diversity index and were dominated by more than 50% of one genera of either *Escherichia *spp. or *Enterococcus *spp. In neonate p8, p20, p22 and p27, multiple bacterial genera were present with no single genus contributing with more than 30% of total bacteria (Figure 3). The differences in diversity could not be explained or correlated to clinical characteristics like NEC score, number of days with antibiotics, time of surgery, or gestational age.

**Table 4:** The consensus tags from the 16S rRNA gene library.

Genus/family	class	typestrain	Sequence
Corynebacterium	Actinobacteria	10-27	CTTAACGCAGCAAGTCGAACGGAAAGGCCCAAGCTTGCTTGGGTACTCGAGT

Acidimicrobium	Actinobacteria	7-54	CTTAACGCAGCAAGTCGGCAAGCGGGTGCGTAACACG

Actinomycetales	Actinobacteria	C50	AACGATGAAGCCCAGCTTGCTGGGTGGATTAGTGGCGAAC

Bifidobacterium	Actinobacteria	C35	AACGGGATCCCAGGAGCTTGCTCCTGGGTGAGAGTGGCGAAC

Clavibacter	Actinobacteria	9-66	AATGCATGCAAGTCGAACGGTGATGTCAGAGCTTGCTCTGGCGGATCAGTGGCGAAC

Corynebacterineae	Actinobacteria	B33	AACGGAAAGGCCCTGCTTGCAGGGTGCTCGAGTGGCGAAC

Micrococcineae	Actinobacteria	B54	AACGATGAAGCCCAGCTTGCTGGGCGGATTAGTGGCGAAC

Propionibacterium	Actinobacteria	9-22	AACGGAAAGGCCCTGCTTTTGTGGGGTGCTCGAGTGGCGAAC

Bradyrhizobium	α-proteobacteria	7-95	GCAAGTCGAGCGGGCATAGCAATATGTCAGCGGCAGAC

Caulobacter	α-proteobacteria	5-10	AACGGATCCTTCGGGATTAGTGGCGGACGGGTGCGTAACACGTGG

Rhodobacteriaceae	α-proteobacteria	9-24	AGCGAGGACTTCGGTTCTAGCGGCGGACGGGTGCGTAACACGTGAA

Sphingomonadaceae	α-proteobacteria	A48	CCTAACGCAGCAAGTCGAACGAACTCTTCGGAGTTAGTGGCGGAC

Bacillales	Bacilli	10-49	AATGCATGCAAGTCGAGCGGAGTTGACGAGAAGCTTGCTTCTCGGATGCTTAGCGGCGGA

Enterococcus	Bacilli	A45	AACGCTTCTTTTTCCACCGGAGCTTGCTCCACCGGAAAAGAGGAG

Exiguobacterium	Bacilli	9-61	AGCGCAGGAAGCCGTCTGAACCCTTCGGGGGGACGACGGTGGAATGA

Lactobacillus	Bacilli	6-3	CCTAATGCATGCAAGTCGAGCGAGCGGAACCAACAGATTTACTTCGGTAATGACGTT

Staphylococcus	Bacilli	B55	AGCGAACAGACAAGGAGCTTGCTCCTTTGACGTTAGCGGCGGAC

Bacteroidales	Bacteroidetes/Chlorobi group	5-73	CTTAATACATGCAAGTCGAGGGGCAGCATGGTCTTAGCTTGCTAAGGCTGATGGCGACCG

Porphyromonas	Bacteroidetes/Chlorobi group	7-33	CTTAACACATGCAAGTCGAGGGGCAGCATTATTTTAGCTTGCTAAGATAGAT

Prevotella	Bacteroidetes/Chlorobi group	7-66	TCCTAACGCATGCAAGTCGAGGGGCAGCATGGAAGAAGCTTGCTTCTTCTGATGGCGA

Burkholderiales	β-proteobacteria	9-31	AACGGTAACAGGTCTTCGGACGCTGACGAGTGGCGA

Cupriavidus	β-proteobacteria	A70	AACGGCAGCGCGGGCTTCGGCCTGGCGGCGAGTGGCGA

Ralstonia	β-proteobacteria	5-36	AACGGCAGCATGATCTAGCTTGCTAGATTGATGGCGAGTGGCGA

zoogloea	β-proteobacteria	10-86	AACGGTAACAGGGAGCTTGCTCCGCTGACGAGTGGCGA

Anaerococcus	Clostridia	6-29	AACGATGAAACTTAATTGATTTCTTCGGAATGATTTTAAGTGGATTAGTGGCGG

Clostridium	Clostridia	C1	AGCGATGAAGCTCCTTCGGGAGTGGATTAGCGGCGGAC

Dialister	Clostridia	A7	AACGGGAAGAGATGAAGAGCTTGCTCTTTATCGAATCCAGTGGCAAAC

Faecalibacterium	Clostridia	C42	AACGGAGTTGAGAGGAGCTTGCTTTTCTTGACTTAG

Finegoldia	Clostridia	10-63	AACGGGATTTAGTAGACAGAAACCTCGGTGGAAGATTACTAATGAGAGTGGCGAACGGGT

Lachnospiraceae	Clostridia	A96	AACGAAGCGATTAAGAGGAAGTTTTCGGATGGAATCTTAATTGACTGA

Papillibacter	Clostridia	C16	AACGGAGCACCTTGAAAGAGACTTCGGTCAATGGATGAGACTGCTT

Peptoniphilus	Clostridia	7-94	AGCGATGAAATTTTGACAGATCCCTTCGGGGTGAAGATAAAATGGATTAGCGGCGGA

Ruminococcaceae	Clostridia	9-81	AACGGAGTTAATTTTGTTGAAGTTTTCGGATGGATACGAAGTTAACTTAGTGGCGA

Veillonella	Clostridia	9-73	AACGGACAGATAGAGAGCTTGCTCTCTTGAAGTTAGTGGCGAAC

Cystobacteraceae	Δ-proteobacteria	7-10	AACGCAGCAAGTCGGGTGCGTAACACGTGGG

Acinetobacter	γ-proteobacteria	9-10	AACGCAGCAAGTCGAGCGGAGATGAGGTGCTTGCACCTTATCTTAGCGGCGGAC

Enhydrobacter	γ-proteobacteria	A49	AACGATGATTATCTAGCTTGCTAGATATGATT

Enterobacteriaceae	γ-proteobacteria	C5	AACGGTAGCACAGAGAGCTTGCTCTCGGGTGACGAGTGGCGGAC

Pseudomonas	γ-proteobacteria	C19	AGCGGTAGAGAGAAGCTTGCTTCTCTTGAGAGCGGCGGAC

Rickettsiella	γ-proteobacteria	A22	CCTAACGCAGCAAGTCGAACGGCAGCACAGTAAAGATTTCGGTCTTTAT

Shigella/Escherichia	γ-proteobacteria	C26	AACGGTAACAGGAAACAGCTTGCTGATTTGCTGACGAGTGGCGGAC

The individual tags (N = 364) were assigned to the closest mono-phylogenetic group.

**Figure 4:**
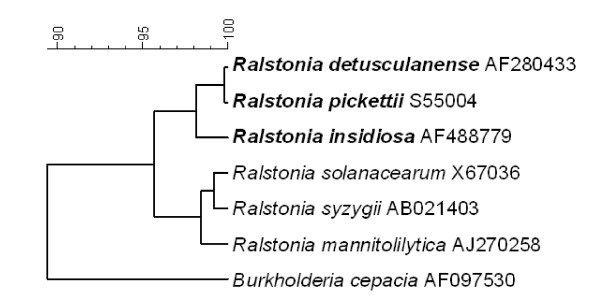
**Phylogenetic relationship among *Ralstonia *detected in the tissue samples from the NEC infants**. *R. detusculanense*, *R. pickettii *and *R. insidiosa *did all have more than 99% similarity with the matched *Ralstonia *tag from the 16S rRNA gene clone library from this study. The bacteria names and the accession numbers are shown.

The bacteria associated with the tissue in the individually neonates have the potential to reveal bacterial pathogens related to the pathogenesis of NEC. In the *δ-proteobacteria *group *Escherichia/Shigella *genera dominated with a frequency of 45% out of all *δ-proteobacteria *and were present in 5 of the 8 neonates with an average frequency of 24% (±36%). The Enterobacteriaceae group consisted of virtually one tag but it was similar to genera of *Citrobacter, Enterobacter *(*Klebsiella*) and *Erwinia *and was detected in 4 of the neonates. The taxonomic class *Clostridia *contained 10 different tags belonging to a variety of different genera (Table 4), the two most prominent being *Clostridium *and *Anaerococcus *detected in four and three neonates, respectively. A tag matching the potential pathogen *Finegoldia *was found twice in two different neonates. One of the specimen characterised histologically exhibiting pneumatosis intestinalis was also observed to include the genus *Clostridium*. The most prevalent tag belonged to *Ralstonia *being present in 7 out of 8 neonates, with an average of 9% (±5%). *R. detusculanense, R. pickettii *and *R. insidiosa *were revealed with more than 99% similarity (Figure 4).

## Discussion

The establishment of the microbiota early in life and the symbiosis with the human gastrointestinal tract has important consequences for human health and physiology. The interactions can have beneficial nutritional, immunological, and developmental effect or even pathogenic effects for the host [13-16]. In this study the bacterial composition has been characterised for the first time directly on tissue samples from neonates with fulminate NEC. The specimens were collected from a single neonatal hospital unit with a consistent treatment and a similar environment over a period of 6 years. Even though, the study is naturally limited in number of patients this is the first description done in situ and not on surrogates in the form of faecal samples or experimental animals. FISH combined with laser capture microdissection ensured that only bacterial DNA from lumen and mucus was sampled and that no contaminations from the surrounding material or environment could occur. Furthermore, cloning and pyrosequencing used here has previously been shown to be efficient for the characterization of the intestinal microbiota [17-19]. The presence of bacterial colonization in the small intestine and large intestine was documented and visualized by a general bacterial FISH probe and this method has previously been used to reveal bacterial spatial distribution in the intestine of experimentally colonised animals [20,21]. In general, tissues with disease were heavily colonised by bacteria but we could not correlate the bacterial colonisation to NEC-score, days with antibiotics or type of antibiotics nor type of nutrition. This colonization might be because of resistance to or wrong choice of antibiotics or because the antibiotics do not reaches the bacteria because of stop of blood supply. It has recently been shown that antibiotics do not clear gut microbiota in neonates but reduce the diversity of bacterial species [22]. We were therefore interested in finding which bacterial groups that colonized the surgical removed tissues.

The dominance of *Proteobacteria *could explain the susceptibility of preterm neonates to NEC or as a course of the antibiotic treatments that all neonates received in this study. From the 16S rRNA gene library the δ-proteobacteria was dominated by *Escherichia*-like organisms and to a lesser extent with *Enterobacteria*. It has previously been described by Wang et al. [18] that δ-proteobacteria dominated the bacterial composition in faecal samples from neonates with NEC but they also found a lower Shannon diversity for NEC patients compared to the control group [18]. This could have been due to the antibiotic treatments. In this study there was no difference in the bacterial composition or Shannon diversity index after long term antibiotic administration (>10 days) compared to less than two days of antibiotic treatments. Furthermore, no difference in bacterial composition was found regardless of the type of antibiotics used for treatment, in contrast to the antibiotic selection seen by Gewolb *et al. *[23]. In general, a very high variation was observed in the bacterial composition of the specimens as well as different diversity indexes and if it had been possible to analyse more samples it would perhaps had been possible to correlate the different variables with the colonization. The colonization of the preterm intestine could have been speculated to be very homogeneous since the neonates were at the same hospital unit (environment) even though Palmer et al., [17] showed that the composition and temporal patterns of the microbial communities in stool samples from term babies varied widely from baby to baby for their first year of life. However the composition of the intestinal microbiota in healthy pre- or term neonates present in the small intestine is not yet known due to the lack of samples [17,18,24,25].

Previous studies based on culture techniques have focused on single organisms as predisposing for NEC [7,26,27]. *Clostridium spp*. and especially *C. perfringens *due to the fermentation of carbonhydrate substrates to hydrogen gas has been suspected [3,6,9]. Very few neonates were colonised with *Clostridium spp*. in this study but there was a significant correlation between a positive signal from the probes for *Clostridium spp *and pneumatosis intestinalis as verified by histopathology. It was specified that this Clostridium colonization was due to *C. butyricum *and *C. parputrificum*. A previous study has shown that these two lactose fermenting clostridium species can induce cecal NEC-like lesions in a gnotobiotic quail model and these lesions may be linked to short-chain fatty acid production [28]. There was no correlation with pneumatosis intestinalis found by X-ray and *Clostridium spp*. and maybe pneumatosis intestinalis described on X-ray is different from the pneumatosis intestinalis described on tissue surgically removed. It seems therefore like *C. butyricum *and *C. parputrificum *are responsible for pneumatosis intestinalis when verified by histopathology, but because of the low frequency of *Clostridium spp *in our samples we believe that the pneumatosis intestinalis is a secondary effect of NEC and that these Clostridia are not the primary pathogens of NEC.

*Ralstonia *and *Propionibacteria *were detected in most of the specimens where laser capture microdissection was used. *Ralstonia *spp. is a new genus including former members of *Burkholderia *spp. (*Burkholderia picketti *and *Burkholderia solanacearum*). *Burkholderia *spp. has been described in children suffering of NEC [29] and *Ralstonia picketti *has been reported to be a persistent Gram-negative nosocomial infectious organism [30]. *R. picketti *can cause harmful infections and is mainly considered as an opportunistic pathogen of little clinical significance but *R. pickettii *isolates have been reported to be resistant or had decreased susceptibility to aminopenicillins, ureidopenicillins, restricted-spectrum cephalosporins, ceftazidime, and aztreonam [31]. The major conditions associated with *R. picketti *infection are bacteraemia/septicaemia and respiratory infections/pneumonia. The bacteria has been isolated from patients diagnosed with Crohn's disease and cystic fibrosis from multiple sides including sputum, blood, wound infections, urine, ear swabs and nose swabs, and cerebrospinal fluid [30,32,33]. Diversity in an ecosystem is important in establishing and preventing dominance by a single pathogenic species. In the samples with *Ralstonia spp*. there were a relatively high diversity of different bacteria and if *Ralstonia *had had a primary effect we would expect a higher dominance of *Ralstonia *and a lower bacterial diversity. Therefore, we cannot conclude from this study whether *Ralstonia *has any effects, on the development of NEC and further studies have to elucidate this or/and if Ralstonia sp. was present because of a higher resistance to the antibiotic treatment.

*Propionibacterium spp*. have previously been described in faecal specimens [17,34]. The presence of this genus has been reported to be the second largest on the adult body and predominant in sebaceous sites [35]; it has probably been found in neonates' small intestine because of skin contact between the mother and the neonate. The reason why it has not been found in higher densities in many other gastrointestinal studies of the microbiota is a general underestimation of *Actinobacteria *created by the choice of primers and a dilution effect in faeces [17].

## Conclusion

This study emphasized the possibility to examine the microbial composition directly on excised human tissues to avoid biases from faecal samples or culturing. Although a large variability of bacteria was found in most of the analyzed specimens, no single or combination of known potential pathogenic bacterial species was dominating the samples suggestive NEC as non-infectious syndrome. However there was a general high presence of *Proteobacteria *and *Ralstonia sp. *which may be due to the antibiotic treatment that all neonates received in this study and a significant correlation between the finding of *C. butyricum *&*C. paraputrificum *and the few histological pneumatosis intestinalis found in this study.

## Methods

### Patient characteristics and sample collection

The study was done retrospectively on neonates with NEC hospitalised from January 2001 to December 2005. All neonates were hospitalised at a single level III Neonatal Intensive Care Unit (NICU) at Rigshospitalet, Copenhagen, Denmark. All neonates had surgical intervention and samples of removed tissue were formalin-fixed and paraffin-embedded at the Department of Pathology, Rigshospitalet. The study was subjected to ethical review and approved by the Ethical Committee for Copenhagen and Frederiksberg, Denmark (KF 01 268923). Patient's records were reviewed in order to characterise the clinical findings, disease progression and clinical outcome. The data gathered from each infant were: gestational age, weight at birth, onset of symptoms, feeding prior to onset of NEC, antibiotic usage prior to operation of NEC, and outcome.

### NEC disease evaluation (NEC-score)

Unfortunately, there is no standard pathological characterization of NEC. We decided to characterize the tissue macroscopic from the characterization made by the pathology that originally looked at the tissue and histologically after haematoxylin and eosin (HE) staining. All histologically samples were independently evaluated by two trained pathologists; at the Department of Pathology, Rigshospitalet and at The National Veterinary Institute, Technical University of Denmark.

#### Macroscopic evaluation

Perforation was noted not scored, hemorrhagic mucosa +/- necrotic areas, score 5, pneumatosis intestinal score 5. Amount of tissue <10 cm score 1, 10-30 cm score 2, >30 cm score 3.

#### Histology evaluation

The formalin-fixed and paraffin-embedded samples were sectioned 3 μm, mounted on slides and stained with HE. The HE slides were graded as follows: (A) Necroses volving; a) luminal epithelia, b) whole mucosa, c) submucosa, d) tunica muscularis; (B) Vascularity; a) oedema, b) bleeding, c) micro-thrombing, d) haemosiderine, (C) Inflammation; a) unspecific (granulocytes), b) eosinophils, c) vasculitis, d) pseudomembranes, e) granulation tissue, f) granulomas, g) granulomas, h) fibrosis, i) atrophy 1)mucosa 2) all other layers; e) and f) was not included in the score but used to graduate the tissue in acute or chronic NEC. (D) Various, 1) ganglion cells 2) non-ganglion cells. All histopathological characteristics were scored one except (D) that was used to distinguish NEC from Hirschsprung's disease. The *NEC-score *score is the addition of the macroscopic evaluation and the histology evaluation

### Bacterial detection by 16S rRNA in situ Hybridization on Formalin-Fixed Tissue Sections

Paraffin was removed of the tissue sections with xylene and dehydrated in 96% ethanol for 30 min. All specimens were hybridized with both a general bacterial probe EUB338 and with selective probes. Probes were synthesized at Eurofins MWG Operon (Ebersberg, Germany) and described in Table 1. Two probes (S-S-C.paraputri-181 and S-S-C. butyricum-663) were designed in ARB http://www.arb-silva.de in this study. The probes were approved for their specificity to closest bacterial type strains by an in silico probe search in RDP release 10 http://rdp.cme.msu.edu/, and experimental verified for signal intensities and specificity by FISH targeting pure culture of *C. butyricum *CCUG4217^T^; *C. paraputrificum *CCUG32755^T^; *C. difficile *ATTC17857 and *C. perfringens *NCTC8449 injected into a piece of pig lung treated as the rest of the tissue samples.

Hybridization was done in 20 μl of hybridization buffer (100 nM Tris, pH 7.2. 0.9 M NaCl, 0.1% sodium dodecyl sulphate) added 100 ng of probe at 45°C for 16 h in a humidified chamber. Slides were washed in 100 ml of preheated (37°C) hybridization buffer for 15 min and subsequently in 10 ml of preheated (37°C) washing solution (100 mM Tris, pH 7.2, 0.9 M NaCl) for 15 min. Slides were rinsed in water and air-dried. All slides were scored as follows: 0) no or low density of bacteria, 1) moderate density of bacteria, 2) high density of bacteria.

### NEC tissues used for Laser Capture Micro dissection

Eight intestinal tissue samples were included. The microdissection was performed on tissues excised from 4 neonates that were treated with antibiotics less than 2 days and from 4 neonates treated with antibiotics 10 days or more before surgery. Three μm sections of the tissues were cut (knife was changed between cuts) and mounted on the 0.17-mm PALM^® ^POL-membrane slides (P.A.L.M. Microlaser Technologies AG, Bernried, Germany) and kept at 4°C until use. The slides were hybridized with bacterial probes as previously described.

### Laser Capture Microdissection

A PALM Robot-Microbeam system (P.A.L.M. Microlaser Technologies AG) consisting of an Axivert 200 M microscope (Carl Zeiss, Oberkochen, Germany) equipped for fluorescence with a 100-W Hg lamp, a 40x/1.30 oil Fluar objective (Carl Zeiss), filter set XF53 (Omega Optical, Brattleboro, VT, USA) and the PALM RoboSoftware version 1.2 (P.A.L.M Microlaser Technologies AG) was used. Bacteria were visualized by FISH using the general bacterial probe EUB338 and dissected from both the intestinal lumen and mucus of the surgical tissue by the cutting and catapulting function, RoboLPC as previous described [12]. The micro-dissected area from the lumen and mucus associated tissues were never in contact with any external contaminators because the micro-dissected area is cut by a laser and "transported" to the tube by a photonic force and against gravity as described by Carl Zeiss AG, Deutschland http://www.zeiss.de/. The risk for external contaminators is therefore minimal.

The catapulting material was collected in the cap of a 200 μl Thermo-Tube (ABgene, Epsom, UK) containing 20 μl proteinase K buffer. The microdissected material was digested in proteinase K buffer (10 mM Tris-HCl, pH 8.0, 150 mM NaCl, 10 mM EDTA, 0.1% sodium dodecyl sulphate, 1 U proteinase K) at 55°C for 72 h. Subsequently, the proteinase K was inactivated at 95°C for 15 min. Two μl of solution were subsequently used as template for the polymerase chain reaction (PCR).

### Clone library and sequencing of intestinal bacteria

The primers Bact64f and Bact109r1 (Eurofins MWG Operon ) were used for 16S rRNA gene amplification of the hyper variable region V1 from the small subunit ribosomal RNA gene (Table 1). PCRs (always including a non template control) were done in 20 μl volumes containing 1 × PCR buffer [20 mM Tris-HCl (pH 8.4) and 50 mM KCl], 200 μM dNTP, 500 nM each primer, 3.3 mM MgCl, and 1 U of Pfu DNA polymerase (Invitrogen Corporation, Carlsbad, CA), which creates blunt end fragments. The thermal profiles were as follows: an initial denaturation step at 94°C for 3 min; 30 cycles of 94°C for 30 s, 50°C for 30 s, and 72°C for 30 s; and a final elongation step at 72°C for 5 min. The amplicons were purified by using phenol/chloroform (P/C, pH 8.0), and the DNA was precipitated with 2.5 M ammonium acetate in ethanol. After two washes with 80% (v/v) ethanol, the DNA pellet was dried and resuspended in 10 μl, 0.2 μl filtrated, double-distilled water. Following the manufacturer's descriptions the cloning was done by using a Zero blunt TOPO cloning kit (Invitrogen Corporation). Fifty to hundred colonies from each cloning were picked and sequenced by pyrosequencing. A PYROMark Q96 ID was used to short DNA sequencing of the approximately 40-60 bp clone insert using the recommended protocol (Biotage AB, Uppsala, Sweden) as described previously using the primer PyroBact64f [19]. The sequences (tags) were imported into the software BioNumerics 4.61 and manually checked, aligned and filtered for high quality sequences. Sanger sequencing with an Applied Biosystem 3130 Genetic Analyzer (Foster City, CA, USA) was used to check consensus tags for the pyrosequencing accuracy. The Sequence match analysis tool in the Ribosomal database project 10 http://rdp.cme.msu.edu/ was used to assign the Phylogenetic position of each consensus tag. The search criteria were for both type and non-type strains, both environmental (uncultured) sequences and isolates, near-full-length sequences (>1200 bases) of good quality. If there was a consensus at the genus level the tag was assigned this taxonomic classification. If no such consensus was found, the classification proceeded up one level to family and again if no taxonomic affiliation could be assigned the tag continued to be proceeded up the tree as described by Huse et al., [36]. In some cases it was not possible to assign a domain and these sequences might represent new novel organisms or the sequences might be biased, in these cases the tags were excluded from the dataset. In total 364 sequences were finally included in the alignment.

The phylogenetic analysis was done by downloading 16S rRNA gene sequences longer than 1,200 base pair from the RDP database of the *Ralstonia *type strains http://rdp.cme.msu.edu. The RDP alignment was used and a phylogenetic tree was constructed by using the Ward algorithm in the software Bionumerics. *Burkholderia cepacia *(GenBank accession no. AF097530) was used as an out-group.

### Statistics

The statistical analysis was done in two steps: First, the association between one predictor at a time and the NEC score was analysed by robust least squares methodology adjusting for gestational age. This is equivalent to a normal linear GEE modal with working independence correlation structure on child level. For each predictor the estimated change in expected NEC score is reported with Wald 95% confidence limits in parentheses. The overall association between the predictor and the NEC score is evaluated by a robust score-test.

Second, we formulate a normal linear GEE model including gestational age and all predictors with a robust score-test p-value below 0.1 in the above analyse. This multivariable model is then reduced by backwards elimination using 0.05 as a cut-off level.

All analyses were performed using PROC GENMOD in SAS version 9.1 (SAS Institute, Cary, NC).

## Authors' contributions

BS carried out the data mining from the hospital studies, carried out the in situ studies and participated writing the manuscript. SB; MB and KAK participated in the design of the study and coordination and helped to draft the manuscript. PLP and TKJ performed the histopathology of the samples and scored the degree of NEC in each tissue sample. CP did the statistical analysis. JK participated in collecting the samples. LM carried out the sequencing and sequence analysis and participated in writing the manuscript. All authors read and approved the final manuscript.
